# Critical role of Wuhan cabin hospitals in controlling the local COVID-19 pandemic

**DOI:** 10.1017/ice.2020.167

**Published:** 2020-04-22

**Authors:** Wenlong Yao, Xueren Wang, Tianzhu Liu

**Affiliations:** Department of Anesthesiology, Tongji Hospital, Tongji Medical College, Huazhong University of Science and Technology, Wuhan, China

*To the Editor—*COVID-19 is quickly spreading all over the world. The total number of confirmed cases has exceeded 1.6 million in just 2 months.^1^ Patients with a variety of respiratory symptoms have flooded into hospitals in a relative short time, posing an enormous challenge to every healthcare system. Wuhan was the first center of the pandemic, and it had the highest number of cases in China. But the pandemic in Wuhan was controlled by 2 months of lockdown beginning January 23, 2020, and newly detected cases of COVID-19 have now decreased to zero. Among a series of preventive approaches,^2^ cabin hospitals played a critical role in isolating mild and asymptomatic cases. Here, we evaluate the role of cabin hospitals in controlling the COVID-19 pandemic by retrospectively analyzing the correlation between available beds in cabin hospitals and epidemic data.

We obtained the data regarding total daily beds available in cabin hospitals from the official website of the Wuhan municipal government, and we extracted daily numbers of newly diagnosed cases, newly cured cases, and new deaths, and we calculated the overall recovery rate and mortality from COVID-19 in Wuhan from the official website of the National Health Commission of the People’s Republic of China. COVID-19 cases were diagnosed according to history, symptoms, chest CT, and nucleic acid test.^3^ From February 12 to February 14, a clinical diagnosis of COVID-19 was applied to make sure that every patient received immediate treatment in Wuhan. Therefore, the number of cases diagnosed in these 3 days dramatically increased, and we was excluded these data from our analysis. We used SPSS version 19.0 software (IBM, Armonk, NY) for the statistical analysis. A Pearson correlation analysis was performed by correlating cabin beds with all epidemic data. *P* < .05 was considered a significant difference.

The official government website reported a total of 28 designated hospitals with 8,254 beds for COVID-19 patients in Wuhan before February 4, 2020. The utilization ratio of beds was as high as 99.1%. On February 4, 2020, the first cabin hospital in Hongshan stadium opened with 1,000 beds. By February 26, 2020, a total of 17 cabin hospitals with 35,499 beds had been set up in Wuhan; overall these cabin hospitals received ~12,000 mild cases of COVID-19. The final utilization ratio of cabin beds was ~33.8%. All epidemiological data and their fluctuating trends with the increase in cabin beds are shown in Figure 1.

**Fig. 1.**
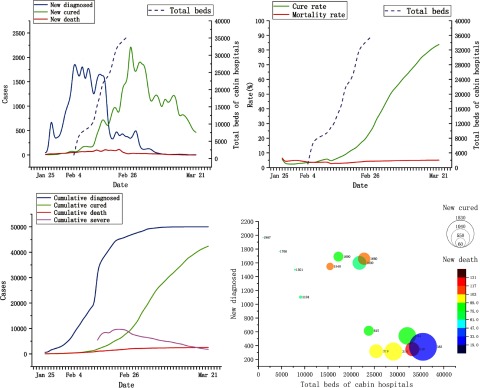
The relationships between total beds of cabin hospitals and epidemic data of COVID-19 in Wuhan. Data were obtained from National Health Commission of China and people’s government of Wuhan to Mar 22, 2020.

By statistical analysis, the number of newly diagnosed cases showed a highly negative correlation with the availability of cabin beds (r = −0.833; *P* < .0001). We detected a highly negative correlation between the number of new death cases and the number of cabin beds (r = −0.859; *P* < .0001). The overall recovery rate was positive correlated with cabin beds (r = 0.961; *P* < .0001). In addition, we detected a significantly decrease of severe cases in the hospital with the increase of cabin beds (r = −0.977; *P* < .0001). [A correlation coefficient of 0.8–1.0 indicates a high correlation; 0.6–0.8 indicates a strong correlation; 0.4–0.6 indicates a moderate correlation; 0.2–0.4 indicates a weak correlation; and 0.0–0.2 indicates a very weak or no correlation.]

The approaches for prevention and control of COVID-19 can vary from city to city. However, the principle of controlling contagious diseases is to isolate the source of infection, to cut off transmission, and to protect vulnerable populations.^4^ Although both COVID-19 and SARS are respiratory diseases caused by coronavirus, COVID-19 differs from SARS^5^ in that many mild and asymptomatic cases of COVID-19 also have transmissibility, and these cases are often missed and not isolated. Therefore, the management of mild or asymptomatic COVID-19 cases is equally important as the treatment of severe cases. Our analysis showed that, with the increase of available beds by cabin hospitals, the newly diagnosed cases and severe cases decreased. Thus, the cabin hospitals played an important role in controlling the COVID-19 pandemic. They effectively prevented family infection or community spread. Early treatment of mild cases can prevent COVID-19 cases from deteriorating.

Cabin hospitals were mainly responsible for the treatment of mildly ill patients. All admitted patients were diagnosed by a positive nucleic acid test, concern regarding cross infection was alleviated. In these temporary hospitals, patients were also cared for by professional medical staff. When a case became severe, the patient was transferred to a designated infectious hospital immediately. Food, accommodation, medication, and examination were paid by the government. These incentives greatly increased the motivation of mildly ill patients to be admitted to cabin hospitals, which reduced social mobility and the risk of community infection. At the same time, timely medical treatment also improved prognoses, avoiding exacerbation of the disease.^6^ In addition, initiation of cabin hospitals reduced the workload of designated infectious hospitals, so the limited public medical resources could be used to treat severe patients and thus reduce the death rate.

According to Xu et al,^7^ the cost of cabin hospitals was low enough that the government could support the roll out on a large enough scale to ensure rapid sequester of cases. Short-term training should be employed to equip cabin hospital staff with self-protection and medical care. Psychological counseling for patients and medical staff should be provided to alleviate anxiety and panic. We also advocate communication and entertainment activity between patients. Online visits for comprehensive mental consultation were also available.

A cabin hospital is like a large community clinic. Home quarantine and community isolation play an important role in the treatment of mild cases, but there is a risk of neglecting some cases, which could lead to community transmission, and a percentage of patients become severely ill. In Wuhan, cabin hospitals connected traditional community clinics and hospitals to achieve early diagnosis, timely treatment, and effective isolation of COVID-19 patients. In conclusion, these cabin hospitals were an important part of effectively controlling the COVID-19 pandemic in Wuhan.

## Supplementary material

For supplementary material accompanying this paper visit http://dx.doi.org/10.1017/ice.2020.167. click here to view supplementary material
