# Supplementary material for: Critical role of Wuhan cabin hospitals in controlling the local COVID-19 pandemic
Source: Infect Control Hosp Epidemiol. 2020 Apr 22:1–2. doi: 10.1017/ice.2020.167 (PMC7200851; doi:10.1017/ice.2020.167)
Supplement: Supplementary file 1 [file S0899823X20001671sup001.docx]

Figure 1: The relationships between total beds of cabin hospitals and epidemic data of COVID-19 in Wuhan. Data were obtained from National Health Commission of China and people's government of Wuhan to Mar 22, 2020.
